# Exploring the multiparameter nature of EUV-visible wave mixing at the FERMI FEL

**DOI:** 10.1063/1.5111501

**Published:** 2019-07-29

**Authors:** L. Foglia, F. Capotondi, H. Höppner, A. Gessini, L. Giannessi, G. Kurdi, I. Lopez Quintas, C. Masciovecchio, M. Kiskinova, R. Mincigrucci, D. Naumenko, I. P. Nikolov, E. Pedersoli, G. M. Rossi, A. Simoncig, F. Bencivenga

**Affiliations:** 1 Elettra Sincrotrone Trieste S.C.p.A., S.S. 14 km 163.5 in Area Science Park, I-34149 Basovizza, Trieste, Italy; 2 Institute for Radiation Physics, Helmholtz-Zentrum Dresden-Rossendorf e.V., 01328 Dresden, Germany; 3 Physics Department and The Hamburg Centre for Ultrafast Imaging, University of Hamburg, Luruper Chaussee 149, 22761 Hamburg, Germany

## Abstract

The rapid development of extreme ultraviolet (EUV) and x-ray ultrafast coherent light sources such as free electron lasers (FELs) has triggered the extension of wave-mixing techniques to short wavelengths. This class of experiments, based on the interaction of matter with multiple light pulses through the *N*th order susceptibility, holds the promise of combining intrinsic ultrafast time resolution and background-free signal detection with nanometer spatial resolution and chemical specificity. A successful approach in this direction has been the combination of the unique characteristics of the seeded FEL FERMI with dedicated four-wave-mixing (FWM) setups, which leads to the demonstration of EUV-based transient grating (TG) spectroscopy. In this perspective paper, we discuss how the TG approach can be extended toward more general FWM spectroscopies by exploring the intrinsic multiparameter nature of nonlinear processes, which derives from the ability of controlling the properties of each field independently.

## I. INTRODUCTION

The nonlinear optical response of matter arises from the interaction of strong electromagnetic fields through the *N*th order susceptibility of a material, in what is called an (*N *+* *1) wave mixing process. Since only sufficiently strong fields can interact nonlinearly with matter, the development of nonlinear optics (NLO) as an experimental tool required the invention of a source of intense coherent light: the laser. The practical relevance of NLO was clear shortly after. Indeed, the interaction of matter with N electric fields, each of frequency *ω_i_*, momentum **k**_*i*_, and polarization ${\hat{n}}_{i}$, can act as the source for an additional field of different frequencies, momenta, and polarizations. Moreover, the possibility of controlling properties such as intensity, wavelength, angle of incidence, polarization, or arrival time for each field independently gives rise to a manifold of different experimental techniques and technologically relevant applications. These methods are nowadays well established at optical and near-infrared wavelengths in diverse fields of science, in particular for the spectroscopy of fundamental excitations of matter with high specificity. However, the optical wavelengths of table-top lasers (>250 nm) impose constraints for accessing the nanometer spatial resolution typical of ultrafast molecular dynamics. Additionally, the low photon energy of a few eV does not allow to access core resonances, which would provide chemical specific excitation, and limits the probed interactions to vibrational and valence band excitations.

**FIG. 1.**
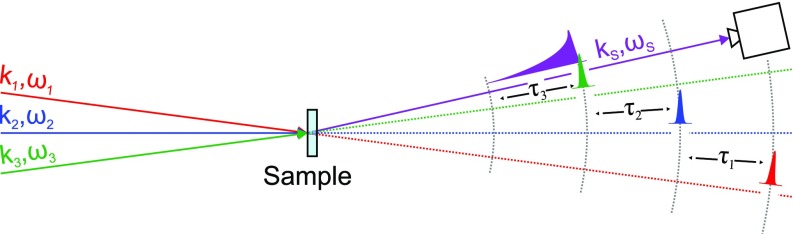
Scheme of the most general time-resolved FWM experiment. Three pulses of frequencies *ω*_1_, *ω*_2_, and *ω*_3_ and momenta **k**_1_, **k**_2_, and **k**_3_ separated by time delays *τ*_1_ and *τ*_2_, respectively, interact on the sample to generate a signal beam of frequency *ω_S_* and momentum **k**_*S*_ that evolves over time *τ*_3_.

The combination of the intrinsic multiparameter nature of wave-mixing with the chemical selectivity of light-matter interactions at extreme ultraviolet (EUV) and X-ray photon energies holds the promise of leading to a unique class of experiments, suitable to monitor on fs-timescale and nm-lengthscale dynamic processes, charge and energy transfer, and correlations between atoms in a material.^1–8^ Moreover, these short wavelengths offer a much larger spatial resolution or, in other words, allow us to access a larger momentum range in reciprocal space, e.g., for the investigation of collective dynamics and transport properties at the nanoscale.^9–14^ Similar to what occurred in the visible regime, the extension of NLO to high photon energies required the development of novel light sources, capable of delivering EUV and X-ray pulses of unprecedented brightness and coherence: the free electron lasers (FELs). Nevertheless, the efforts in this direction remain pioneering and are limited to the demonstration of a given process under specific experimental conditions. This way, the literature reports the proof of principle, among others, of parametric downconversion,^15^ stimulated and amplified spontaneous emission,^16,17^ two-photon absorption,^18^ second harmonic generation,^19,20^ and X-ray/VIS wave mixing.^21–23^ Most of these experiments are based on second order processes, limited to systems with broken inversion symmetry in the visible and EUV photon energy range, and thus, despite being more efficient, have an intrinsically smaller range of applicability than the third order, or four wave mixing (FWM), interactions. FWM phenomena include, besides all conventional “pump-probe” spectroscopies, also the most common noncollinear IR-VIS NLO techniques such as stimulated Raman scattering, transient grating (TG), or 2D spectroscopy.^24,25^ Here, three distinct coherent beams interact on the sample to generate a signal beam of frequency *ω_S_* and wavevector **k**_*S*_ dictated by energy and momentum conservation laws, the so-called phase matching condition,^24,25^ as depicted in Fig. 1. The pulses are separated by time delays *τ*_1_ and *τ*_2_, and the signal is emitted over time *τ*_3_. Thus, depending on the controlled time delays, the experiments can access both populations and coherences between excited states.

A decade long theoretical vision notwithstanding,^1,2,4,5^ the implementation of FWM experiments at high photon energies is hampered by fundamental reasons along with technical difficulties in combining multiple short-wavelength input pulses. Indeed, far from electronic resonances, the third order nonlinear susceptibility scales as the inverse of the frequency squared for each of the incoming beams, causing a significant decrease in the cross sections when moving from visible to EUV photon energies.^24^ These limitations have been recently overcome in two dedicated setups that were developed and implemented at the FERMI FEL source^26^ with the aim of performing noncollinear EUV FWM experiments based on the TG scheme: the compact EUV-optical wave mixing setup mini-TIMER and the purely EUV-FWM beamline TIMER.^11^ TG experiments are a particular class of FWM where two of the incoming pulses, called the “pump,” have the same frequency and are overlapped in time (*τ*_1_ = 0) on the sample at an angle to generate a transient standing wave that effectively acts as a diffraction grating for the third pulse, the “probe.” The interference pattern of the two pumps excites impulsive modes with momentum $|k|=4\pi \;\text{sin}\;(\theta )/\lambda_{\mathrm{ex}}$, where 2*θ* is the pump crossing angle and *λ_ex_* is their wavelength. The probe is then scattered at $\text{sin}\;\theta_{\mathrm{out}}=\text{sin}\;\theta_{\mathrm{in}}-|k|\lambda_{3}/2\pi$, along a direction that can be chosen to be largely different from the direction of the incoming beams. The emitted signal as a function of *τ*_2_ carries information on the excited population dynamics, frequencies of collective modes, transport times, etc. Exploiting the TG interaction has proven to be a successful strategy for implementing EUV/x-ray NLO since the background free geometry allows the detection of weak signals. A prototypical version of mini-TIMER was used to demonstrate the first EUV-induced TG back in 2015,^22^ while the first purely EUV FWM signal was reported on TIMER in 2018.^27^

In this perspective article, we review the current status of EUV FWM, describing the most recent achievements obtained at FERMI using mini-TIMER and TIMER, and build up on this to discuss the foreseen efforts to exploit the VIS-EUV TG setup for the exploration of multidimensional spectroscopies. Indeed, both these setups already provide the tunability in some of the parameters required to carry on a more general FWM experiment. In particular, the time delay between the pump pulses, *τ*_1_, can be varied to measure the coherence time of the excitation and can be combined with the scanning of the pump-probe delay *τ*_2_ to perform 2D spectroscopies. Additionally, both setups allow the variation of the pump and probe wavelengths, which can be selectively tuned to resonances or used in more complex multicolor wave mixing processes such as coherent Raman scattering.

## II. RECENT ADVANCES OF EUV-BASED FWM

The main difference between the two EUV-FWM setups at FERMI, described in detail elsewhere,^11,28^ is the probe wavelength and consequently the accessible momentum range |k| in TG experiments. Indeed, the optical probe limits the accessible exchanged momentum at mini-TIMER to $|k_{\mathrm{ex}}|<2|k_{\mathrm{opt}}|\approx 0.05\;{\text{nm}}^{-1}$, while TIMER can access |k| values as large as 1 nm^−1^ by using an EUV probe. However, the efficiency of nonlinear interactions is characterized by an unfavorable scaling with respect to the photon frequencies^24^ of the input beams, which can be mitigated by exploiting core resonances and which, in turn, makes the optical probing advantageous for the investigation of purely electronic processes. Therefore, mini-TIMER is not only an ideal setup for testing novel FEL-based wave-mixing schemes but also a complementary setup with respect to TIMER.

Both setups are based on a similar geometrical beam splitting concept, previously tested in optical TG experiments.^29^ The FEL beam is split by the edge of a plane mirror working at grazing incidence, instead of using diffractive optics as it is commonly done in the optical regime. This, on one hand, is to prevent the strong absorption and the high losses of transmission optics in the EUV regime. On the other hand, the direction of the reflected beams and the reflectivity of the mirror do not depend on the FEL wavelength, guaranteeing the achromaticity of the system. The optical probe allows mini-TIMER to be built as a miniaturized setup inside a hosting chamber, focusing the beam upstream with bendable Kirkpatrick-Baez (KB) mirrors and then splitting and recombining the two pump beams at the sample with three planar C-coated mirrors arranged in a parallelogram geometry. The position of each mirror, both parallel and perpendicular to the beam, as well as their pitch and roll can be controlled with encoded motors to allow for a continuous tuning of 2*θ*. The same reliable positioning of the mirrors can be taken advantage of to introduce a continuous and controlled time delay *τ*_1_ between the two pump pulses. TIMER, which aims at exploiting a larger |k| range, requires the TG to be probed by a third FEL pulse of wavevector comparable or larger than the one of the excitation pulses. Therefore, the setup scales to be a whole beamline, where each pulse is focused at the sample by its own toroidal mirror at four fixed crossing angles, thus giving up on the continuous tunability in 2*θ* and on the flexibility of the more compact setup. Nevertheless, parameters such as the delay between the pump pulses or the wavelength of both pump and probe pulses remain tunable, albeit the latter is limited to discrete transmission wavelengths of the delay line.

The mini-TIMER setup described above is a largely improved version of the one used to demonstrate EUV FWM.^22^ Since then, it was used, among others, to generate surface acoustic waves (SAWs) and optical and longitudinal acoustic phonons, detected both in forward and back-diffraction geometries, at frequencies well above the optically accessible ones,^30^ to probe nanoscale thermal transport,^31^ to demonstrate the activation of intraband Auger relaxation processes across the Si L-edge,^32^ and, combined with TIMER, to determine the mechanical and thermal responses of a thin SiC membrane in an extended |k|·b regime, where *b* is the membrane thickness.^33^ Moreover, the improved capability of controlling *τ*_1_ has allowed us to measure the pulse duration, temporal profile, and coherence of the FEL pulses in a cross correlation scheme.^34^

The first purely EUV FWM interaction was demonstrated in 2018 at TIMER exploiting the transient diffraction of FEL harmonics from a TG generated by the fundamental FEL radiation.^27^ This experiment takes advantage of the fact that, due to the short absorption length of EUV light in matter, the EUV TGs can be considered as thin gratings and diffraction can occur at any angle, differently to what typically occurs in the optical or hard X-ray regime. This opens up new experimental strategies for energy resolved FWM approaches, such as the simultaneous generation of TGs at different excitation wavelengths probed by the same beam or, vice versa, the simultaneous probing at two *λ*_3_ of the same TG. Moreover, if combined with the capability of controlling the relative phase between the pulses, these frequency domain schemes could potentially allow us to access fs or even subfemtosecond dynamics. More recently, Bencivenga *et al.*^35^ succeeded in demonstrating the capability of TIMER of investigating atomic dynamics in the mesoscopic wavevector range (0.1–1 nm^−1^), while Monaco *et al.*^36^ investigated the phonon decay time in metallic glasses at $|k|=0.26\;{\text{nm}}^{-1}$.

This rapid overview clearly shows that many of the ingredients for a further development of EUV FWM toward a more general, multiparameter approach are already available in the FERMI setups. In the following sections, we will discuss in additional detail preliminary findings associated with three main parameters: the pump-pump delay *τ*_1_, the probe wavelength, and, finally, the excitation wavelength.

## III. ROLE OF THE PULSE COHERENCE

In a recent article, Capotondi *et al.*^34^ demonstrated how mini-TIMER could be used to characterize the time structure of the FERMI FEL pulses. Our approach was based on the extension of a self-diffraction scheme successfully employed to measure both the pulse duration and the coherence time of a Nd:YAG laser and of a dye laser.^37^ The transient grating cross correlation scheme exploited at mini-TIMER relies on measuring the diffracted probe intensity while scanning the delay between the excitation pulses *τ*_1_, keeping the pump-probe delay *τ*_2_ at a fixed positive value where the response of the material can be assumed to be constant. The latter is to ensure that no jitter in the pump-probe arrival time is reflected in the TG intensity and consequently on the measurements. Similar experiments^37,38^ and related theories^39^ have shown that a correct description of the diffraction efficiency as a function of *τ*_1_, and thus the resulting time-dependent trace, involves fourth-order correlation functions that depend on the ratio of the longitudinal coherence time *τ_c_* and of the pulse length *τ_p_*. In other words, while for transform-limited pulses where *τ_c_* ≈ *τ_p_*, the measured trace reduces to the simple intensity autocorrelation of the pump pulses, for *τ_c_* < *τ_p_*, the measured trace results from the sum of a background given by the intensity auto-correlation times, the *τ_c_*/*τ_p_* ratio, and a “spike” of width ∼*τ_c_*, the so-called coherence spike. It is worth mentioning that preliminary experiments that relied on the interference visibility to measure the spatiotemporal coherence of FEL pulses were performed at the self-amplification of spontaneous emission (SASE) FEL FLASH.^40,41^ These experiments however do not exploit dynamical wave mixing and rely on the observation of the interference fringes on a CCD detector.

**FIG. 2.**
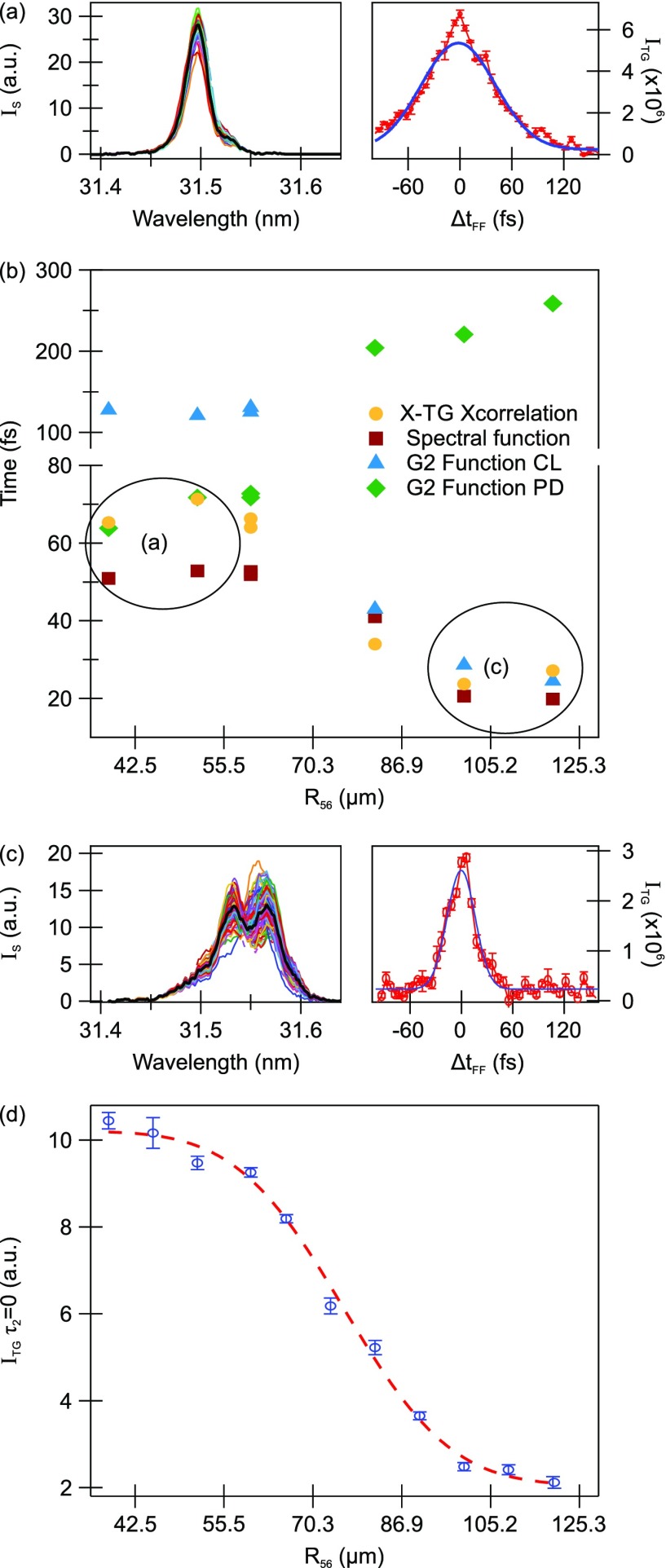
(a) (Left) Transform-limited spectra of FERMI and (right) the corresponding cross correlation trace, in red, with the Gaussian fit (blue). (b) Extracted pulse length/coherence time as a function of the dispersive section R_56_ strength for different evaluation methods: cross-correlation FWHM (yellow dots), spectral function width (red squares), coherence length (blue triangles), and pulse duration (green diamonds) obtained from the second order correlation function G2. (c) (Left) FERMI spectra for a high value of R_56_ and (right) the corresponding cross correlation trace, in red, with the Gaussian fit, in blue. (d) Transient grating intensity at *τ*_2_ = 0 for the different values of the dispersive section: Spoiling the pulse coherence results in almost one order of magnitude decrease.

This capability of TG-based cross-correlation experiments of measuring simultaneously the pulse duration and the coherence time has been used to evaluate the effects of a decreased pulse coherence on the efficiency of FWM processes. Indeed, while EUV FWM experiments were demonstrated at FERMI, experiments have yet to be successfully performed at higher photon energies, where full coherence is not available. The main difference of FERMI compared with X-ray FELs is the seeding process with an external laser, which allows us to control the microbunching of the electron beam, leading to FEL amplification. This results in almost transform-limited FEL pulses that maintain the temporal and spatial coherence properties of the seeding laser.^42^ Indeed, in a seeded high gain harmonic generation FEL such as FERMI, the lasing process is started by the interaction of the seed with the electron bunch in the first undulator, where the beam is longitudinally modulated in energy with the periodicity of the seed laser wavelength. In order to convert this energy modulation into a density modulation that initiates the light amplification in the final FEL amplifier, the beam is then injected into a dispersive section: A magnetic device where, depending on the magnet dispersion (also indicated as the R56 parameter), the electron path length is inversely proportional to the electron energy. The strength of the dispersive section and the seed laser intensity are normally tuned to maximize the FEL output intensity while preserving the longitudinal coherence. However, it has been shown that the temporal pulse shape can be significantly affected, up to a splitting of the FEL pulse into two subpulses,^43,44^ in the case of an excess of dispersion or seed power. This causes an over-bunching, i.e., a nonlinear wave breaking process. When combined to a chirped seed laser, the temporal splitting can be associated with visible changes in the FEL spectral profile and possibly with the overall coherence of the pulse.^45^ Thus, the full control over the seeding parameters in seeded FELs gives access to the tunability of the *τ_c_*/*τ_p_* ratio. FELs based on external seeding are at the moment limited to the EUV–soft X-ray spectral range, while hard X-ray machines are based on the self-amplification of the spontaneous emission (SASE) principle. SASE FEL pulses, as a result of the stochastic origin of the radiation start-up, are characterized by bursts of several short radiation spikes that are correlated neither in amplitude nor phase. As a result, the coherence length of SASE pulses is typically significantly shorter than the pulse length, which could limit the efficiency of nonlinear optical experiments. Indeed, the order of magnitude intensity of a FWM process can be obtained from^24,25^

$$\begin{array}{c} I^{\left(3\right)}(\omega_{s})∼\left[\prod_{i=1}^{3}I_{i}(\omega_{i})\right]\cdot |\chi^{\left(3\right)}{|}^{2}\\ \cdot {\left\{\omega_{s}L\;\text{exp}\;(-L/2L_{\alpha })\text{sinc}(L/L_{c})\right\}}^{2} \end{array},$$ (1)

where *I_i_* is the power of the input field, *χ*^(3)^ is the third order nonlinear optical susceptibility, and *L*, *L_α_*, and *L_c_* are the sample length, the light penetration depth at the signal wavelength, and the coherence length of the process. The latter is directly related to the phase matching conditions and depends on experimental parameters such as the geometry and the coherence of the pulses. Indeed, for transform-limited pulses, the finite pulse bandwidth (Δ*ω* ∝ Δ*t*^−1^) corresponds naturally to a wavevector spread |Δk|∼Δω/c, which is related to the coherence length as $L_{c}=|\Delta k{|}^{-1}$. Therefore, it is expected that for decreasing coherence lengths (or coherence times), the efficiency of the FWM process will rapidly decrease. In order to quantify this dependence of FWM efficiency on the pulse longitudinal coherence, we present novel results from an experiment aiming at measuring both the FEL cross correlation (at positive delays *τ*_2_ > 0) and the TG signal at *τ*_2_ = 0. This has allowed us to relate the efficiency of the FWM process with a decreasing *τ_c_*/*τ_p_* ratio and get indications of possible implications of the limited coherence time in FWM experiments at SASE sources, where the coherence time is mostly limited to the width of an individual SASE spike. The experiment has been performed on a 500 nm thick SiN membrane using the mini-TIMER setup with the FERMI FEL1 source tuned at *λ_FEL_* = 31.45 nm (8th harmonic of *λ_seed_* = 251.6 nm) with a crossing angle 2*θ* = 4.8°, which corresponds to a grating spacing of Λ_*TG*_ = 375.5 nm. The TG diffraction intensity was probed at a fixed delay *τ*_2_ = 1.7 ps by the output of a noncollinear optical parametric amplifier (NOPA) seeded by the FERMI seed laser for users (SLU) and tuned to *λ_probe_* = 525 nm. The left panel of Fig. 2(a) shows a series of FERMI spectra and their average (in black) for a dispersive section strength R_56_ ≃ 53 *μ*m, i.e., when the FERMI pulse is transform-limited, and the right panels depict the corresponding cross correlation. Instead, Fig. 2(c) shows the spectra and cross correlation for R_56_ ≃ 125 *μ*m, corresponding to the overbunching condition. In the reported case, the temporally split pulses also show a clear splitting in the spectral domain due to the residual chirp in the seed laser.^45^ The cross-correlation curve is much narrower and is almost completely dominated by the coherence spike. Panel (b) shows the measured FWHM of the cross correlation curves (yellow dots), the estimated pulse duration from the spectral function assuming a Gaussian pulse shape (red squares) together with the coherence length (blue triangles), and the pulse duration (green diamonds) extracted from the second order correlation function G2.^46–49^ We observe that the G2 function well describes the temporal shape of the pulses either with the coherence length or with the pulse duration, respectively, depending on if the pulses are transform-limited or not, in agreement with the theory by Trebino *et al.*^39^ Panel (d) reports the *τ*_1_ = *τ*_2_ = 0 intensity of the TG signal for each of the applied R_56_ strengths. Clearly, the effect of increasing the dispersive section and thereby spoiling the temporal coherence of the pulses by about a factor of three is to decrease the efficiency of the pulse already by almost one order of magnitude. This would extrapolate, in an order of magnitude estimation, to a decrease by four orders of magnitude in the efficiency, when going from the transform-limited seeded source to a SASE source with spikes of few fs duration. Evidently, this imposes serious constraints in performing TG-like experiments at hard X-ray sources, especially since any temporal jitter comparable with the duration of the single SASE spike (≃1 fs) between the pulses after the splitting would seriously compromise the feasibility. Thus, these experiments would benefit experimental schemes relying on phase gratings, similar to optical phase masks, which are able to split and recombine the pulses with a high degree of temporal and spatial overlap, hence granting an efficient impulsive stimulus.^50^ As an example, Svetina *et al.*^51^ recently demonstrated the ability of generating permanent gratings of hundreds of nanometer spatial periods with ∼3 keV radiation at the Swiss FEL source by exploiting the Talbot effect for converging beams diffracted by phase gratings.^51^ X-ray FWM is definitely one of the research fields that would largely benefit from the numerous attempts of increasing the source coherence which are ongoing at most facilities, for example, self-seeding,^52–54^ high-brightness (or improved) SASE,^55–57^ purified SASE,^58^ or harmonic lasing.^59,60^ All these methods aim at increasing the longitudinal coherence of SASE pulses by spectrally narrowing the emission. While the spectral narrowing can be routinely done using monochromators, it is worth noting that the proposed schemes also further amplify the narrowed emission. However, since all of them still start from the stochastic noise in the electron bunch, none of these schemes is capable of improving intensity and wavelength fluctuations, with the first that can sometimes be as high as 100% RMS.

## IV. EXPLORING THE DEPENDENCE ON THE PROBE WAVELENGTH

The comparison of the first results from mini-TIMER and TIMER shows a clear probe frequency dependence that goes well beyond the effects ascribable to the progressive decrease in the grating spacing. In particular, the most striking effect is the absence of a strong instantaneous response at *τ*_1_ = *τ*_2_ = 0 when probing with an off-resonant EUV pulse^35^ instead of a visible laser pulse. This initial peak, the so-called electronic peak, is related to the direct interaction of the probe with photoexcited electron-hole pairs in the sample and, therefore, is representative of the electronic response. Most recent preliminary results indicate that its disappearance is mitigated when probing at core excitation resonances. Indeed, while visible and resonant EUV probes strongly couple to the changes in the refraction index associated with the presence of free carriers, the off-resonant EUV probe, instead, is much more sensitive to effects related to the changes in total electronic density. Again, this strong difference confirms the complementarity of the two setups and the superior ability of purely EUV-TG in probing the lattice response, i.e., the thermal and density gratings generated after the relaxation of the initial carrier grating. Furthermore, the observed difference in probing on- or off-resonance, if confirmed by systematic studies, would represent a remarkable step toward one of the main goals of EUV wave mixing: the site specific probing of atomic correlations.

**FIG. 3.**
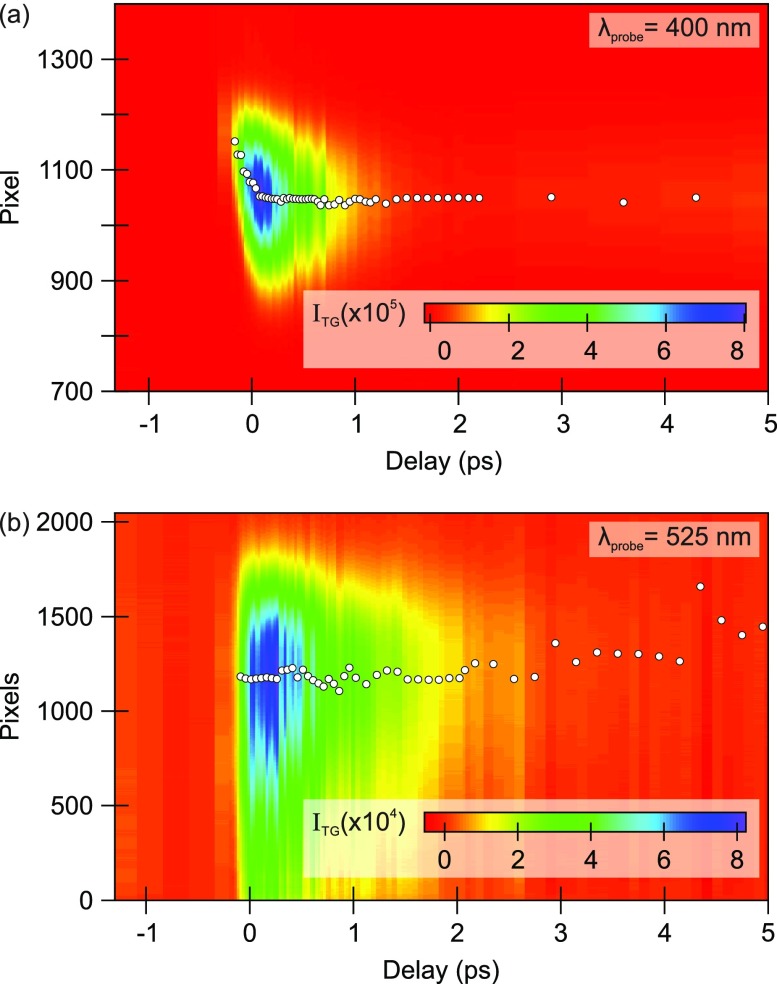
TG signal spectra as a function of the *τ*_2_ delay on a 57 nm thick SiC membrane for (a) a probe wavelength of 400 nm and (b) a broadband probe centered at 525 nm. Please note the significantly broader vertical axis in the latter case. The white dots indicate the position of the spectrum centroid. When probing well above the bandgap energy, the signal spectrum exhibits a significant ultrafast angular shift, attributed to the time-dependent changes perpendicular to the beam propagation direction, which is not observed for the probe at the bandgap edge.

Nevertheless, at visible wavelengths, the probe photon energy can encode relevant information. Indeed, the TG can be seen as a transient local spectrometer, and the angular dependence of the TG signal contains information on the changes of the spectral weight of the diffracted beam. This is observed in panel (a) of Fig. 3, which compares the 2D plots of the TG signal spectrum as a function of time delay *τ*_2_ from a 57 nm thick membrane of SiC for two different probe pulses. Panel (a) shows the standard probe of mini-TIMER at 400 nm (3.1 eV) and panel (b) the diffracted beam when the probe is provided by the output of the NOPA tuned at a central wavelength of 525 nm (2.36 eV). The first probe energy is well above the 2.3 eV bandgap energy of SiC, and one observes a clear change of the diffraction angle for the early time delays. This behavior has been observed on the same SiC membrane also with the EUV probe and has been attributed to nonisotropic TG-induced changes in the refractive index of the material along the sample thickness, i.e., perpendicularly to the grating vector.^33^ The same angular shift, however, is not observed when the probe is tuned to a photon energy right at the bandgap energy, as depicted in panel (b). Moreover, already at a first sight, it is evident that the ten times larger bandwidth of the 6 fs long NOPA output spans, when diffracted, a ten times larger detector area compared with the narrow band standard probe pulse (60 fs FWHM). These preliminary results represent a first attempt at performing multiplexed EUV-TG spectroscopy.^61–64^ Indeed, the setup can be easily improved to effectively exploit the transient grating as a spectrometer by adding a focusing element before the detector and thus improving the spectral resolution via Fourier plane imaging. The capability of performing multiplexed measurements with EUV/X-ray photoexcitation is fundamental for example in the study of complex molecules, where one could excite resonantly at one atomic site and probe specific excitonic or vibrational resonances within a broadband probe.

One experimental scheme that is instead unique to the EUV-induced TGs relies again on the thin grating condition and involves the probing of the same grating with different probe energies, similar to what shown for the EUV probe by Foglia *et al.*^27^ Indeed, instead of using a broadband probe around the Bragg condition, for a relaxed phase matching, one can potentially use discrete and well separated probe wavelengths tuned at different resonances, or below and above the bandgap. This way, each of the diffracted signals would propagate along its own background-free direction and the dynamics can be easily separated, naturally helping the disentanglement of the different responses. This can go as far as, in a feature that to date would be exclusive of the TIMER setup, combining simultaneous visible and EUV probing of sufficiently long-spaced TGs and thereby probe both the population and density gratings separately and selectively.

## V. TOWARD XCRS: MULTI-COLOR EXCITATIONS IN FWM

Undoubtedly, a major step toward the realization of X-ray Coherent Raman Scattering (XCRS) and thus toward the measurement of interatomic correlations is a full understanding of multicolor excitations in high-photon energy wave mixing. Preliminary attempts have been performed at mini-TIMER and TIMER in several multicolor FEL emission configurations where the FEL works either in the twin seed or in the split undulator mode. In the first case,^65^ the machine is seeded by two laser pulses of different frequencies and separated in time such that each one interacts with a different fraction of the electron bunch. Then, each microbunching is amplified at the same harmonic number in the undulators, obtaining two FEL pulses whose energy separation is a harmonic of the seed energy difference and is, in general, limited by the FEL gain to be ∼Δ*λ*/*λ* < 1% and time separation corresponds to the original seed time separation on the electron bunch (Δ*t* = 0.2–0.8 ps). In the second configuration,^66^ instead, the undulator section is split into two parts, and the same seed is amplified at two different harmonics, one for each undulator section. This way, the energy separation between the two can be as large as the difference between the two harmonic numbers times the seed energy, and the two pulses are intrinsically overlapped in time.

A first attempt at a multicolor excitation, with two FEL pulses separated by 160 meV in the twin seed configuration, has shown, besides a possible anti-Stokes signal, a second nonshifted signal bearing several similarities to the TG signal observed in single color excitation.^67^ The exact nature of these signals and how they are related to the machine settings require further investigations and go beyond the scope of this work.

A purely electronic rather than vibrational XCRS experiment would however require larger energy separation between the excitation beams, typically on the order of a few eV. This kind of configuration was attempted on TIMER with a setup similar to the one of Foglia *et al.*,^27^ i.e., giving up on the capability of controlling the delay *τ*_2_ and looking at the scattering of copropagating FEL harmonics. Preliminary results suggest indeed that the transient diffraction pattern of the harmonics is affected by the interaction between the two-color FEL pump pulses. However, the very low electronic scattering cross section of the EUV probe beam, due to the fact that none of the employed photon energies was at resonance with core transitions, was a limiting factor for these experiments where the expected signal is purely of electronic nature.

**FIG. 4.**
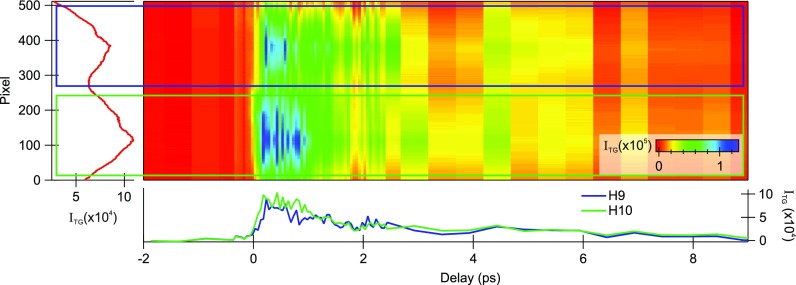
Diffraction of the probe beam from two TGs generated simultaneously using two consecutive harmonics of the FERMI seed laser (H9, blue rectangle and H10, green). The lower panel shows the corresponding time evolution of the signals.

Figure 4 shows a further attempt at combining multiple color excitations. In this case, again, the machine was configured in the split undulator setup at the harmonics 9 and 10 of a 365 nm seed. The two pulses, intrinsically time-overlapped at the source, were geometrically split and recombined with a 5° crossing angle with mini-TIMER on a ZnO crystal and probed in transmission with the output of the NOPA tuned at ∼600 nm. When all four pulses (one of each color per each input direction **k**_1_ and **k**_2_) are time-overlapped on the sample, two TGs of different periodicities are generated, thus diffracting the probe with an angular separation. This would in principle allow us to measure simultaneously and separately the TG signal due to two distinct EUV excitations. In this particular case, since neither of the pump energies nor the probe energy was resonant with any material's transition, no clear differences are observed in the time-resolved traces. However, this experimental setup could be exploited, for example, to measure electronic dynamics simultaneously below and above an absorption edge, where one would predict strong differences in the observed dynamics.^32^ This capability is of particular interest if combined with the multiplexed probe scheme discussed in Sec. IV since it would, for example, allow for the study of molecular excitonic properties for different excitation conditions in a single measurement. Additionally, other interesting effects, such as higher-order wave mixing processes, are expected to occur and might be observed. For example, the two gratings at **k**_1_ and **k**_2_ could “interfere” and diffract the optical beam through the difference grating vector **q** = **k**_1_ – **k**_2_. One can expect the time evolution of this signal to be characterized by modulations at *ν_beat_* = *ν*_1_ – *ν*_2_, due to the beatings of the two high-|k| phonons launched by the two EUV TGs. The (optical) signal at **k**_*out*_ then encodes the signatures of the dynamics of excitations otherwise inaccessible to optical radiation and may provide information, e.g., on the coupling between these distinct excitations. As a fifth-order process, this TG “interference” is obviously much less efficient than the TG. However, extrapolating from the diffraction efficiencies routinely achieved at mini-TIMER, one could expect, in some case, an appreciable diffraction efficiency of 10^−6^ for such a fifth order interaction.

## VI. CONCLUSIONS

In this perspective paper, we have reviewed the current progress of the EUV/X-ray transient grating and discussed the immediately achievable steps toward the extension at these photon energies of more general four wave mixing processes. Clearly, the low efficiency of these processes calls for the high photon fluxes that, to date, can be provided only by FELs. Nevertheless, the signal is still not high enough to avoid long integration times while achieving a sufficient signal to noise ratio. This bottleneck, however, will relax with the advent of high repetition rate sources as European X-FEL and LCLS-II. Nevertheless, on a more fundamental level, we have shown how the efficiency of the process is drastically affected by the coherence of the source, making the seeded FERMI source the ideal facility for performing these kinds of experiments. Indeed, the two dedicated FWM setups available at the facility have proven to be successful tools for performing TG experiments, to the point that they are being employed routinely for external user experiments. Moreover, both their mechanical setup and the photon source already present all the ingredients required for more complex, multidimensional spectroscopies. For example, EUV-based 2D spectroscopies in both the time and the frequency domain could be at reach within a short time frame. Finally, the increased understanding and control of the fundamental physics of high-photon energy wave-mixing slowly lead to the exploration of more exotic, higher-order processes that go beyond any possible expectation until a couple of years ago. These developments, thus, hold great promise to routinely monitor on fs-timescale and nm-lengthscale dynamic processes, charge and energy transfer, and correlations between atoms in a material. This would give access to the study of a large variety of systems that range from technologically relevant nanostructures to large biological molecules and would open the field to an increasingly large scientific community.
